# Genetic characterization and phylogenetic study of Lakor goat from Southwest Maluku Regency based on mitochondrial COI gene

**DOI:** 10.14202/vetworld.2020.1209-1220

**Published:** 2020-06-28

**Authors:** Maman Rumanta, Rony Marsyal Kunda, Slamet Diah Volkandari, Indriawati Indriawati, Pieter Kakisina

**Affiliations:** 1 Department of Biology Education, Study Program, Faculty of Education and Teacher Training, Universitas Terbuka, Jakarta, Indonesia; 2 Department of Biology, Faculty of Mathematics and Natural Science, Universitas Pattimura, Ambon, Indonesia; 3 Research Center for Biotechnology, Indonesian Institute of Sciences, Jakarta, Indonesia

**Keywords:** artiodactyla, CO1 gene, Lakor goat, mitochondrial, phylogenetic study

## Abstract

**Aim:**

This study is aimed at characterizing the genetic and phylogenetic structure of Lakor goats as indigenous livestock from the Southwest Maluku Regency based on mitochondrial COI gene sequences.

**Materials and Methods:**

The genomes of 103 follicle samples from Lakor goats, collected from Lakor Island, were analyzed. The polymerase chain reaction was used to amplify 1548 bp of the mitochondrial COI gene using two primer pairs (COIA and COIB). Following sequencing, genetic variation and phylogenetic relationship were established using MEGA version X software.

**Results:**

The results of multiple COI gene alignment of the total sequences identified four polymorphic nucleotides that function as genetic markers between individual animals within the Lakor goat population. These correspond to positions 228 (A-G), 519 (G-A), 900 (C-T), and 1266 (T-C). Phylogenetic signals based on the COI gene showed that Lakor goat breed is a monophyletic group or single clade with a bootstrap value of 100% by the neighbor-joining (NJ) and maximum likelihood (ML) evolutionary models. This data indicated that evolutionarily, the Lakor goat breed has a very close kinship with three goat breeds from China: The Meigu goat (KM 244714.1), Chinese Tibet (*Capra hircus*) (KJ 940969.1), and *C. hircus* (KP 677510.1). Phylogenetic information based on the cladistics system classified the Lakor goat as a single clade (monophyletic group). The low-genetic diversity within populations indicates that there has been an inbreeding depression occurring at a very high frequency.

**Conclusion:**

We conclude that the Lakor goat may be divided into a single clade or monophyletic group based on the COI gene sequence. Four nucleotides were identified that can be used as genetic markers among individual animals within the Lakor goat population, as well as *C. hircus* and others as derived from GenBank data. The Lakor goat population has a high level of inbreeding depression as a result of geographical isolation, which supports the formation of a monophyletic group with different genetic characteristics, and does not allow the introduction of males from other breeds. Phylogenetic signals indicated that *Capra aegagrus* (bezoar) is the ancestor of the native goats in Indonesia, including the Lakor goats.

## Introduction

The genus *Capra*, which contains domestic goats and their wild relatives (bezoars, turs, markhors, and ibex) displays a uniquely old-world distribution [1]. There are several goat breeds that have adapted to the environment and geography in the territory of Indonesia (Marica, Bengal, Etawah, Kosta, Kacang, Gembrong, Muara, Samosir, and Lakor goats) [2]. Goats are known as livestock that produce meat, milk, and fur, but are also used in traditional and religious ceremonies [3]. The Lakor goat is a domestic goat (*Capra hircus*) with a very high level of adaptation and is categorized as an indigenous goat breed by the Southwest Maluku Regency based on the Minister of Agriculture Decree No. 2912/Kpts/OT.140/6/2011 with a very limited distribution, only on Lakor Island. The Lakor goat is suspected to be a cross-breed between the Etawah goat descendant and the Kacang goat (Indonesian native goat) [4]. The domesticated goat (*C. hircus*) is a very adaptable animal and geographically widely distributed across several continents [5]. Fossil data suggest that the *Capra* species first appeared in Central Asia and that an adaptive species radiation occurred during the Plio-Pleistocene period [6,7]. In addition, Pidancier *et al*. [1] reported that the history of the *Capra* species is poorly understood and it is compounded by the fact that the radiation of the *Capra* taxa apparently occurred rapidly [7], making it difficult to assess the number of species and their phylogenetic relationships [1].

Mitochondrial DNA (mtDNA) is maternally inherited [8,9]. Recently, the mitochondrial genome has been a preferred source for identifying genetic markers and for determining genetic variability to reveal the phylogenetic relationships among populations or species. This is due, in part, to the rapidly evolving methods that use readily obtainable samples from any tissue type [10-12]. The use of molecular techniques to analyze relationships between species and populations has become widespread, and identifying nucleotide sequence variations in mtDNA offer a powerful tool for molecular phylogenetics at various taxonomic levels [13]. MtDNA markers have been widely used for discriminating differences between closely related species [14], and also for the analysis of the genetic structure of a population [10]. Such genetic data are of particular interest because the Lakor goat may reveal molecular evidence of gene flow or genetic isolation, which is undetectable using traditional morphological studies. Studies have shown that features such as maternal inheritance, non-recombination, multiple copies, and a high rate of evolution render mtDNA a strong molecular marker for analyzing intra- and inter-specific relationships when compared with nuclear markers [8,9]. The COI region is now widely used for the molecular evaluation of diversity, as it exhibits a strong potential for identifying cryptic species [15] and improves our understanding of geologic history, the evolution of species, and the archipelago biodiversity concept. In addition, studies have shown that the COI mitochondrial gene, which is responsible for carrying out oxidative phosphorylation, is highly conserved across species [16,17]. The COI gene is a very useful tool for genetic studies and shows a greater nucleotide substitution rate and more variation than other mitochondrial genes [8,18]. In addition, the CO1 gene may lead to a better understanding of the genetic structure, recognition of cryptic species, new invasion, and evolution of other species [9].

The genetic characterization and phylogenetic study of Lakor goats using the COI gene have greatly improved our understanding of this breed and its habitat, enabling breeding measures to be designed appropriately. Another important aspect is that the molecular genetics of this goat breed have not been explored throughout the population. This is important as the population of the Lakor goat breed continues to decline significantly each year. The results of this study will be useful for improving our understanding of indigenous livestock breeding strategies in Indonesia. The focus of this research is to characterize the genetic and phylogenetic features of the smallest unit of life that can be identified as monophyletic. Discovery of the mitochondrial COI gene as a standard reference for the systematic studies of animals has transformed molecular genetics by providing a platform to expeditiously find novel lineages and elucidate the phylogeny of ruminants [19].

## Materials and Methods

### Ethical approval

The study does not require ethical approval.

### Sample collection

**Figure-1:**
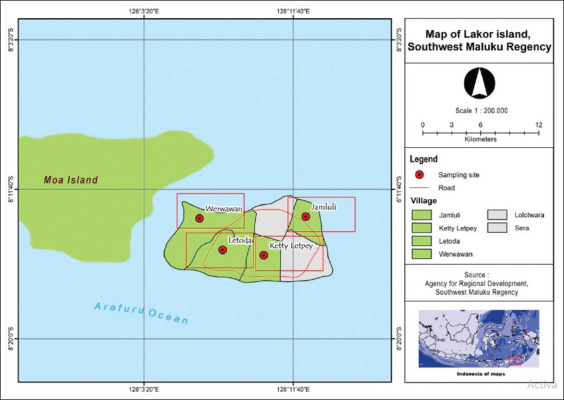
Sampling sites in Lakor Island, Southwest Maluku Regency [Source: Agency for Regional Development, Southwest Maluku Regency, Indonesia].

A total of 103 samples from Lakor Island in Southwest Maluku Regency (Figure-1) were collected from May to June 2018 from four locations: Ketty Letpey (28 samples), Werwawan (20 samples), Yamluli (26 samples), and Letoda (29 samples). Hair follicles from goat tails were collected and stored in envelopes to keep dry. DNA was extracted using a DNA isolation kit (gSYNC^™^ DNA Extraction Kit, Geneaid) and stored at ‒20°C until further examination.

### Primer design

Amplification of the target DNA fragments was done by the polymerase chain reaction (PCR) using two primer pairs, COIA (807 bp) and COIB (1077 bp), and the total size analyzed for the COI gene was 1884 bp. Primers were designed using the Primer3 program (http://www-genome.wi.mit. edu/cgi.bin/primr3.cgi/results_from-primer3) and the mitochondrial genetic sequence data of *C. hircus* mitochondrion (KP662714.1). The two primer sequences utilized for the COI gene amplification were COIA-F: 5′OACAGGACTTGGTAAAAAGAGG-3′Cand COIA-R: 3′n ATACTTCAGGGTGTCCAAAG-5′. The COIB primer sequences were: COIB-F: 5^′-^GACCGAAACCTAAACACAAC-3′ and COIB-F: 3′-CCTAGTTGTATGGGGTATGC-5′ with predicted melting temperatures of 55.1°C and 55°C, respectively.

### Sequencing and phylogenetic analysis

The sequencing of the purified PCR products was done precisely by 1^st^ base sequencing INT (Singapore) and the analysis of the sequences was done using the MEGA program version X [18]. DNA forward and reverse sequences of the COI gene were aligned with Clustal W. Subsequently, the sequences were first edited, followed by multiple alignments with the sequence data related to *Capra aegagrus* mitochondrion (KT290893.1); *C. hircus* mitochondrion (KP662714.1); *Capra caucasica* isolate MA4105 (NC_020683.1); *Capra pyrenaica* (NC_020625.1); *Capra nubiana* COX1 gene (NC_020624.1); *Capra ibex* COX1 gene (NC_020623.1); *Capra falconeri* COX1 gene (NC_020622.1); *Capra sibirica* COX1 gene (NC_020626.1); *C. hircus* from East and Northeast India (KX845672.1); *C. hircus* in China (KP677510.1); Meigu goat (*C. hircus*) (KM244714.1); Chinese Tibetan goat (*C. hircus*) (KJ940969.1); Qaidam Cashmere goat (*C. hircus*) (MG603753.1); Nepalese goat (*C. hircus*) (KY523510.1); Britain and Ireland goat (KY564264.1); *C. hircus* isolate G778 (AB735775.1); Sardinian goat (KJ192226.1); Meriz goat (MH165339.1); and *C. hircus* mitochondria (AB736122.1). Distance analysis was conducted using the NJ tree with bootstrap analysis, which was constructed from 1000 replicates based on Kimura 2-parameter distance using *Ovis aries* (EF490453.1); *Ovis ammon* (KX609626.1); *Ovis canadensis* (JN181255.1); *O. aries* breed Altay (KP981378.1); *O. aries* (KP702285.1); *O. aries* isolate Hamm breed Hamdani (MF004244.1); *Cervus nippon* hortulorum (GU457433.1); *Muntiacus putaoensis* (NC_036430.1); and *Axis porcinus* (MF435989.1) as outgroups. The phylogenetic tree of the samples and the other goat sequences from GenBank were assembled based on the COI gene sequences to establish relationships between the species and the clusters between individual animals.

## Results

### Variation of nucleotide sequences and genetic diversity in the Lakor goat population

A total of 103 DNA samples were isolated and used as a template for COI gene amplification by PCR. The results of PCR for COIA (807 bp), COIB (1077 bp), and the nucleotide variations within the Lakor goat population are provided in Table-1. Multiple alignment of the COI gene resulted in a total sequence of 1548 bp and polymorphic nucleotides were identified that may function as genetic markers between individual animals within the Lakor goat population. The results of the COI gene nucleotide analysis revealed four polymorphism sites between individuals within the population (Table-1). Table-1 also shows that the genetic diversity within the Lakor goat population is considered to be very low and this is likely due to reduced inbreeding. The four polymorphic sites clearly distinguish between the Lakor goat breed, *C. hircus* (KP677510.1), and *C. hircus* Qaidam cashmere (MG603753.1). The four nucleotide changes were: (1) Position 228 (A-G) found in Yamluli goat 1; (2) site 519 (G-A) found in goat 8, Yamluli goat 1, Yamluli goat 2, Yamluli goat 3, and Yamluli goat 8; (3) site 900 (C-T) found in Werwawan goat 8, and Yamluli goat 2; and (4) site 1266 (T-C) found in Werwawan goat 8, Yamluli goat 2, Yamluli goat 3, and Yamluli goat 8. The four nucleotide changes that underwent mutation are categorized as substitutions (transition type).

**Table-1:** Nucleotides variation of Lakor goat based on the COI gene sequence. Identification with the first sequences, and nucleotides identical is denoted by a dot.

Samples		Origin (village)	Polymorphic nucleotides			

			2	5	9	1
			2	1	0	2
			8	9	0	6
						6
Lakor goat 1		Ketty letpey	A	G	C	T
Lakor goat 2		Ketty letpey	.	.	.	.
Lakor goat 3		Ketty letpey	.	.	.	.
Lakor goat 4		Ketty letpey	.	.	.	.
Lakor goat 5		Ketty letpey	.	.	.	.
Lakor goat 6		Ketty letpey	.	.	.	.
Lakor goat 7		Ketty letpey	.	.	.	.
Lakor goat 8		Ketty letpey	.	.	.	.
Lakor goat 1		Werwawan	.	.	.	.
Lakor goat 2		Werwawan	.	.	.	.
Lakor goat 3		Werwawan	.	.	.	.
Lakor goat 4		Werwawan	.	.	.	.
Lakor goat 5		Werwawan	.	.	.	.
Lakor goat 6		Werwawan	.	.	.	.
Lakor goat 7	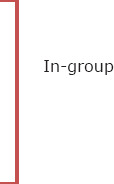	Werwawan	.	.	.	.
Lakor goat 8		Werwawan	.	A	T	C
Lakor goat 1		Yamluli	G	A	.	.
Lakor goat 2		Yamluli	.	A	T	C
Lakor goat 3		Yamluli	.	A	.	C
Lakor goat 4		Yamluli	.	.	.	.
Lakor goat 5		Yamluli	.	.	.	.
Lakor goat 6		Yamluli	.	.	.	.
Lakor goat 7		Yamluli	.	.	.	.
Lakor goat 8		Yamluli	.	A	.	C
Lakor goat 1	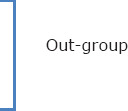	Letoda	.	.	.	.
Lakor goat 2		Letoda	.	.	.	.
Lakor goat 3		Letoda	.	.	.	.
Lakor goat 4		Letoda	.	.	.	.
Lakor goat 5		Letoda	.	.	.	.
Lakor goat 6		Letoda	.	.	.	.

The nucleotide distance estimation within the Lakor goat is shown in Table-2. The nucleotide distance estimation between individual animals within populations was very small and ranged between 0 and 3 nucleotides. These data suggest that there has been a significant amount of inbreeding depression within populations. This is supported by the fact that males with strong genetic quality have not been introduced to Lakor Island, so the breeding process that occurs in the population takes place naturally and has the potential to result in inbreeding depression. A comprehensive strategy is needed to reduce inbreeding depression to increase the performance quality of this breed. Distance estimation of a population is an inherent character in the evolutionary history of organisms.

**Table-2:** Matrix of pairwise sequences diversity (p) of mitochondrial COI gene of Lakor goat.

[1	2	3	4	5	6	7	8	9	10	11	12	13	14	15	16	17	18	19	20	21	22	23	24	25	26	27	28	29	30	31	32	33].	
[1]																																	
[2]		4																															
[3]		4	0																														
[4]		4	0	0																													
[5]		4	0	0	0																												
[6]		4	0	0	0	0																											
[7]		4	0	0	0	0	0																										
[8]		4	0	0	0	0	0	0																									
[9]		4	0	0	0	0	0	0	0																								
[10]		4	0	0	0	0	0	0	0	0																							
[11]		4	0	0	0	0	0	0	0	0	0																						
[12]		4	0	0	0	0	0	0	0	0	0	0																					
[13]		4	0	0	0	0	0	0	0	0	0	0	0																				
[14]		4	0	0	0	0	0	0	0	0	0	0	0	0																			
[15]		4	0	0	0	0	0	0	0	0	0	0	0	0	0																		
[16]		4	0	0	0	0	0	0	0	0	0	0	0	0	0	0																	
[17]		5	3	3	3	3	3	3	3	3	3	3	3	3	3	3	3																
[18]		4	2	2	2	2	2	2	2	2	2	2	2	2	2	2	2	3															
[19]		5	3	3	3	3	3	3	3	3	3	3	3	3	3	3	3	0	3														
[20]		4	2	2	2	2	2	2	2	2	2	2	2	2	2	2	2	1	2	1													
[21]		4	0	0	0	0	0	0	0	0	0	0	0	0	0	0	0	3	2	3	2												
[22]		4	0	0	0	0	0	0	0	0	0	0	0	0	0	0	0	3	2	3	2	0											
[23]		4	0	0	0	0	0	0	0	0	0	0	0	0	0	0	0	3	2	3	2	0	0										
[24]		4	0	0	0	0	0	0	0	0	0	0	0	0	0	0	0	3	2	3	2	0	0	0									
[25]		4	2	2	2	2	2	2	2	2	2	2	2	2	2	2	2	1	2	1	0	2	2	2	2								
[26]		4	0	0	0	0	0	0	0	0	0	0	0	0	0	0	0	3	2	3	2	0	0	0	0	2							
[27]		4	0	0	0	0	0	0	0	0	0	0	0	0	0	0	0	3	2	3	2	0	0	0	0	2	0						
[28]		4	0	0	0	0	0	0	0	0	0	0	0	0	0	0	0	3	2	3	2	0	0	0	0	2	0	0					
[29]		4	0	0	0	0	0	0	0	0	0	0	0	0	0	0	0	3	2	3	2	0	0	0	0	2	0	0	0				
[30]		4	0	0	0	0	0	0	0	0	0	0	0	0	0	0	0	3	2	3	2	0	0	0	0	2	0	0	0	0			
[31]		4	0	0	0	0	0	0	0	0	0	0	0	0	0	0	0	3	2	3	2	0	0	0	0	2	0	0	0	0	0		
[32]		2	4	4	4	4	4	4	4	4	4	4	4	4	4	4	4	5	4	5	4	4	4	4	4	4	4	4	4	4	4	4
[33]		1	3	3	3	3	3	3	3	3	3	3	3	3	3	3	3	4	3	4	3	3	3	3	3	3	3	3	3	3	3	3	1
**Analysis ---------------------------- Distance Estimation**																																	
**Scope ------------------------------- Pairs of taxa**																																	
[1] Capra hircus breed Qaidam cashmere (MG603753.1)																																	
[2] Lakor goat 1 (Ketty Letpey village)																																	
[3] Lakor goat 2 (Ketty Letpey village)																																	
[4] Lakor goat 3 (Ketty Letpey village)																																	
[5] Lakor goat 4 (Ketty Letpey village)																																	
[6] Lakor goat 5 (Ketty Letpey village)																																	
[7] Lakor goat 6 (Ketty Letpey village)																																	
[8] Lakor goat 7 (Ketty Letpey village)																																	
[9] Lakor goat 8 (Ketty Letpey village)																																	
[10] Lakor goat 1 (Werwawan village)																																	
[11] Lakor goat 2 (Werwawan village)																																	
[12] Lakor goat 3 (Werwawan village)																																	
[13] Lakor goat 4 (Werwawan village)																																	
[14] Lakor goat 5 (Werwawan village)																																	
[15] Lakor goat 6 (Werwawan village)																																	
[16] Lakor goat 7 (Werwawan village)																																	
[17] Lakor goat 8 (Werwawan village)																																	
[18] Lakor goat 1 (Yamluli village)																																	
[19] Lakor goat 2 (Yamluli village)																																	
[20] Lakor goat 3 (Yamluli village)																																	
[21] Lakor goat 4 (Yamluli village)																																	
[22] Lakor goat 5 (Yamluli village)																																	
[23] Lakor goat 6 (Yamluli village)																																	
[24] Lakor goat 7 (Yamluli village)																																	
[25] Lakor goat 8 (Yamluli village)																																	
[26] Lakor goat 1 (Letoda village)																																	
[27] Lakor goat 2 (Letoda village)																																	
[28] Lakor goat 3 (Letoda village)																																	
[29] Lakor goat 4 (Letoda village)																																	
[30] Lakor goat 5 (Letoda village)																																	
[31] Lakor goat 6 (Letoda village)																																	
[32] Capra aegagrus (KT290893.1)																																	
[33] Capra hircus mitochondrion (KP662714.1)																																	

### Phylogenetics and phylogeographics of the Lakor goat based on COI sequence

**Figure-2:**
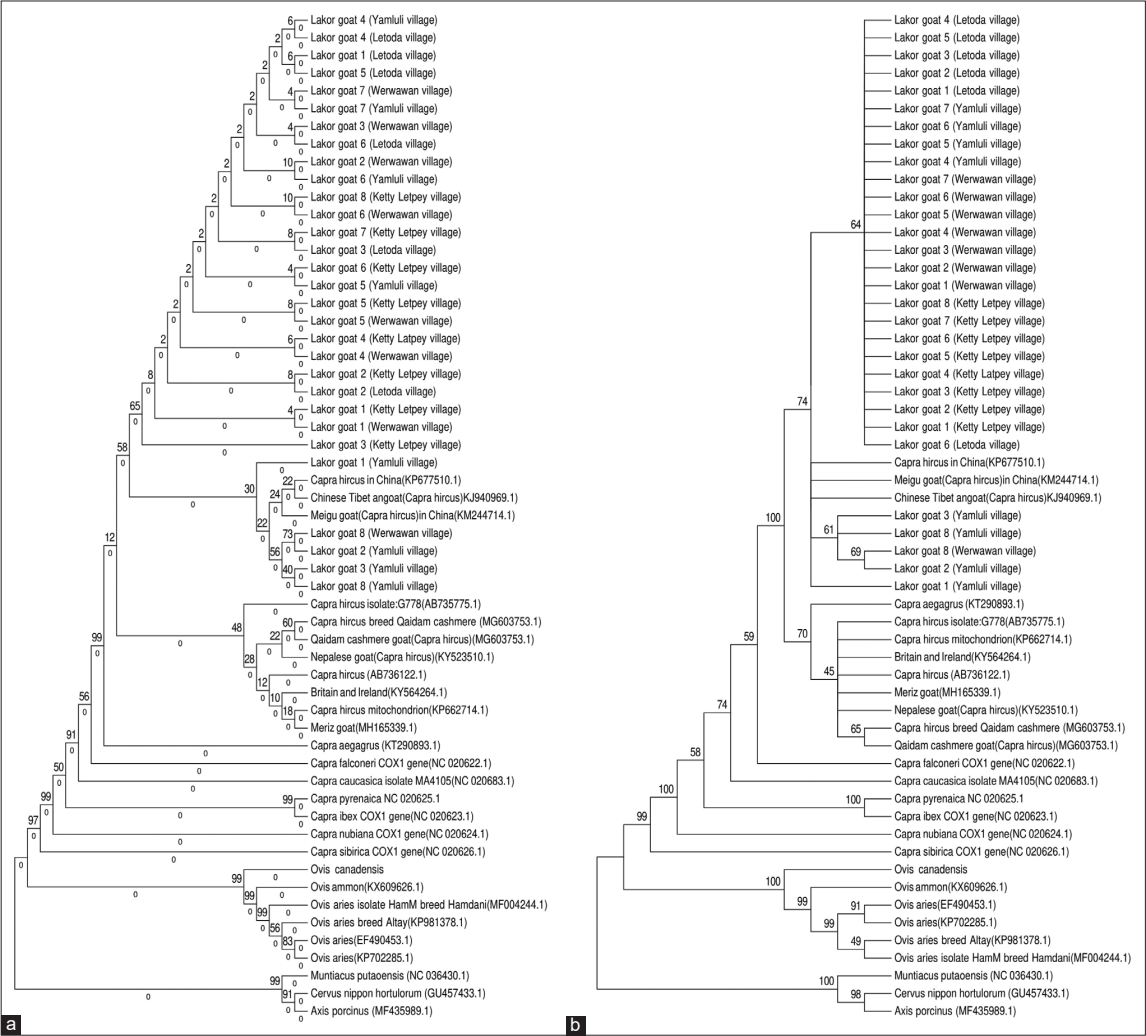
Phylogram of Lakor goat based on maximum likelihood (a) and neighbor-joining (b) methods.

The examination of the phylogenetic relationship of the Lakor goat samples and other related *C. hircus* species was done within the nucleotide range 1-1548 of the COI gene based on the limited data available from GenBank. The taxon identification phenogram of the samples was analyzed by constructing a phylogenetic tree using the NJ and ML methods. Figure-2 shows that the phylogenetic tree of the Lakor goat based on the COI gene nucleotide sequences. The phylogram signal from the COI gene sequence for comparison at the species level is very strong as evidenced by the bootstrap values of 100% in the evolution (NJ and ML) models. The consistency of these two evolutionary models showed that the COI gene has the ability to separate the intra- and inter-species level associated with the phylogram. The phylogenetic signal based on the COI gene showed the Lakor goat breed as a monophyletic group or single clade with a bootstrap value of 100% (Figure-2). All individual animals that were used to design this phylogenetic hierarchy formed a single clade with a genetic distance of 0.0% (Figure-2). This data indicated that evolutionarily, the Lakor goat breed has a very close kinship with three goat breeds from China: The Meigu goat (KM 244714.1), Chinese Tibetan goat (*C. hircus*) (KJ 940969.1), and *C. hircus* (KP677510.1). Two evolutionary models (NJ and ML) revealed that the three goat breeds from China share strong hereditary synapomorphic characteristics with the Lakor goat breed. Phylogenetic information based on the cladistics system classified the Lakor goat as a single clade (monophyletic group) with a genetic distance of 0.0%, which indicates that genetic diversity between individuals within a population is very small. A monophyletic status in the cladistics system with a genetic distance value of 0.0% indicated that an inbreeding process has occurred with a very high frequency within the population. Evolutionarily, a high percentage of breeding in a population will result in a decline in genetic quality and it is correlated to the performance of the population members. The cladistic status that clearly distinguishes the Lakor goat from other goat breeds is thought to be due to the geographical isolation that supports the formation of a monophyletic group with different genetic characters.

## Discussion

### Genetic variation of Lakor goats based on COI gene sequences

Several factors in the habitat indicate that the Lakor goat breed has a very high adaptability because it has survived despite low feed quality, extreme climatic pressures (summer is more harsh), and has developed high resistance to parasitic infections and disease. Salamena *et al*. [4] reported that the Lakor goat breed has the potential to maximally develop at a low cost for the purpose of achieving livestock food security. The optimal development of the Lakor goat should be supported by studies of the genetic resources to design regulations regarding the development of this breed as an indigenous livestock. The present study identified four nucleotides that may be used as genetic markers to distinguish between individual animals within the Lakor goat population. These include positions 228 (A-G), 519 (G-A), 900 (C-T), and 1266 (T-C) of the COI gene (Table-1). These four markers were identified from COI gene sequence alignments from the Lakor goat breed samples along with GenBank sequences of other goats. This genetic characterization technique provides a rapid method to identify and discover new species. Its success depends on the reciprocal monophyly of species and on the strength of an established “barcode gap,” which is a clear delimitation between intra- and inter-specific sequence variability in target genes sequences [20].

Changes in nucleotide composition result from both transversion and transition and can be used as a genetic markers to study natural populations [10,21,22]. Nucleotide substitutions resulting from either transversion or transition can provide a clear understanding of the evolutionary and geographic history of a population [21,22]. Not all genes can be used as genetic markers, and only genes with certain features may be used for genetic characterization and phylogenetic studies [23]. A low intra-individual genetic diversity (0-3 nucleotides) observed within a population indicates that inbreeding depression has occurred in the population. It is thought that the mutation of four nucleotides within the mitochondrial COI gene is the result of genetic compression caused by adaptation to a dry tropical environment. The COI gene is one of three mtDNA encoded subunits of respiratory complex IV. Complex IV is the third and final enzyme of the electron transport chain involved in mitochondrial oxidative phosphorylation. It catalyzes the reduction of oxygen to water to generate the electrochemical proton gradient across the inner mitochondrial membrane that powers the production of ATP. This enzyme utilizes four electrons from the positively charged P side (outside) of the membrane and four protons from the negatively charged N side (inside) for the reduction of dioxygen to form two water molecules [24].

Ayied and Zaqeer [25] studied polymorphisms in the COI gene and its association with milk production and the growth of lambs before weaning in Iraqi Awassi sheep. This study concluded that milk production, birth weight, and weaning weight were not significantly associated with polymorphisms in the COI gene. However, a COI gene polymorphism was significantly associated with weaning weight. As a result, the COI gene was recommended as a genetic marker to genetically improve weaning weight in the Lakor goat, though it showed low genetic variation among individual animals (within the breed). Cox and Hebert, Wares and Cunningham [26,27] demonstrated that the COI gene has a rapid evolution rate to allow discrimination of not only closely associated species but also phylogeographic groups within a single species. In some cases, traditional (conventional) human selection of certain goat breeds slowly results in extreme size differences and unique adaptive mechanisms to manage environmental stresses as illustrated with Lakor goats [28] and large-sized Shire horses [29]. This has an impact on the occurrence of inbreeding depression within natural populations. It is important to realize that local adaptation and artificial selection by humans and/or nature do not always result in a decrease in genetic variation or diversity function within a livestock population.

### Molecular phylogeny and phylogeography of the Lakor goat based on COI gene sequences

Ruminants, being very diverse in their ecology, behavior, physiology, and phylogeographical distribution, represent a subject of interest to many biologists. The diversity among them can be systematically studied using phylogeny. In general, phylogeny relies on variations at the morphological or molecular level to deduce the evolutionary relationship between taxonomic groups. Molecular phylogeny utilizes inheritable structural or functional biomolecular data for constructing phylogenetic trees. A well-resolved phylogenetic tree can describe the relationship, population history, and evolutionary dynamics of a species. Even though the previous studies have proven the efficiency of the COI gene as a genetic marker, a comprehensive phylogenetic analysis has not been performed on ruminants using COI gene sequences. Thus, to represent the inter-relationships between different ruminant species and to reaffirm the efficiency of the COI gene marker, the COI gene sequences of 27 ruminant species were retrieved from GenBank and analyzed in this study. Phylogenetic analysis revealed a single distinct major clade using two evolutionary models (NJ and ML) (Figure-2).

The phylogenetic tree indicated that the Lakor goat shares a kinship with three other goat breeds from China: The Meigu (KM 244714.1), Chinese Tibetan (*C. hircus*) (KJ 940969.1), and *C. hircus* (KP677510.1) goats. Recently, the evolutionary relationship and systematic classification of the Bovidae family remain in dispute and different classification systems exist for bovids based on their phyletic relationships. Morphological and molecular examination has suggested that the Bovidae family should be considered as a monophyletic group, where all the descendants evolved from a common ancestor to form a clade. In contrast, paraphyletic grouping of the Bovidae family, in which most of the descendants of a common evolutionary ancestor form a group excluding a few descendants which form a separate group, has been described in several publications [30].

According to Mason [31], *C. aegagrus* (bezoar) is the direct ancestor of the domestic goat and *C. falconeri* (Markhor), which is a wild goat that has contributed to a number of goat species in Asia. Based on complete Cyt b gene sequences, Indonesian goats have similar genetics as *C. aegagrus*, but many differences exist with *C. falconeri*. This proves that *C. aegagrus* (bezoar) is the ancestor of the native goats of Indonesia [32]. According to Pakpahan *et al*. [32], our phylogenetic tree (NJ and ML) analysis apparently represents two distinct lineages between C. *falconeri* and *C. aegagrus*. The results indicate that *C. aegagrus* (bezoar) is the ancestor of the native goats of Indonesia (Figure-2), and evolutionarily, is also the ancestor of the Lakor goat, which has undergone a process of adaptation in different environments over time. The NJ and ML tree topology clearly shows the close evolutionary relationship between the historical Lakor goat and other goats from GenBank (Figure-2). The Lakor goat is a cross-breed between the Etawah goat descendant and the Kacang goat (Indonesian native goat), but the Etawah goat is genetically dominant [4]. The results of this study indicate that domestic goats in Indonesia originate from one species: The Kacang goat. This is demonstrated by the similarity in phenotype. All Indonesian goat breeds lead to the Kacang goat phenotype. Domestic goats have adapted to their environment over a long period, which leads to some genetic changes [32].

The dispersion of goats across the Asian Continent is prolific, and every country contains goat livestock in which that livestock exhibits few phenotypic differences because it has long adapted with its environment. Indonesia goats form a group with the Laos and Thai goats, which indicate that the Indonesian goats have a genetic distance that is very close to these animals [32]. A number of studies have reported that Asian domestic goats mostly originated from a single ancestor, *C. aegagrus* [33,34]. In addition, Chen *et al*. [35] reported that the Middle East has two types of wild goats (*C. aegagrus and C. ibex*) that may have contributed to the origin and evolution of Asian domestic goats. These two goats can still be found in Tibet and Inner Mongolia.

Disclosure of the phylogeny and phylogeography of livestock is a priority for the design of sustainable conservation strategies and to maximize the use of these species. Animal genetic diversity results from wild ancestors, mutations, genetic drift, and natural and human selection. Only a small part of the diversity that exists in parental species has survived in modern domestic livestock. However, the diversity of domesticated livestock is constantly changing through the randomization of genes in each generation, mutation, and cross-breeding or mixing of the gene pool. This is the basis for the large gains in output achieved from commercial breeds for the identification of genetic erosion due to uncontrolled inbreeding depression, local breeds provide clearer information. In addition to the loss of population data, a major weakness of the current monitoring system is the identification of genes that have genetic clogging or the loss of local breeds due to uncontrolled inbreeding depression. This is an issue that breeding experts should consider as a major threat to genetic diversity of livestock. To establish a comprehensive picture, detailed information is needed regarding the geographical location of local breeds, the distribution of individual members of imported breeds, and genetic material flowing within the gene pool. The COI gene has clearly distinguished different ruminant species and it can serve as an efficient tool for the identification of ruminants as well as the systematic organization of the species into various taxa [36]. Similarly, Ali *et al*. [37] reported that COI gene sequences can distinguish the native goat breeds of Pakistan from the exotic goat breeds.

### The impact of inbreeding on endemic species of the Archipelago

According to Volkandari *et al*. [28], there is low genetic variation within the Lakor goat population after few generations of selection and domestication. This condition is due to improper breeding mechanisms and broodstock management. Conventional breeding practices lead to a loss of genetic where the contribution of parent is practically unknown. Moreover, this practice makes it difficult for selection of the next generation, where the relationships and genetic lineages of the resulting offspring are unclear. This will lead to inbreeding which, in turn, contributes to a loss of genetic variation within the Lakor goat population. The reduction of genetic variations within these populations has been recorded and has also occurred in other ruminants species [38-41]. Breeding programs often use small numbers of broodstock and this practice is primarily responsible for the loss of genetic variation [42,43]. When the number of broodstock is low, there is a high chance of inbreeding, and the resulting contribution to genetic variation is low. Over time, this practice may lead to low genetic variation within a population as demonstrated in this study. However, with proper practice, mitigation of genetic loss may be achieved. In our study, we have found that although genetic variation in populations is reduced, it is important to reduce genetic loss so that it is not significant. In the near term, while waiting for policies related to the use of genetic information from this breed, controlling breeding mechanisms and broodstock management may be done by selecting males from neighboring villages and not from the same village as females. This approach must be taken because on Lakor Island, respective villages have a permanent fence composed of rocks (local name is *Lutur*) to protect the mixing of livestock between villages. We assume that, in this way, the rate of genetic loss in the Lakor goat population can be reduced. This is very important for the traditional livestock industry on Lakor Island and to maintain the future sustainability of this endemic species. If no mitigation measures are taken, the loss of genetic variation in the population may result in severe effects in the future.

Keller and Waller [44] reported that the levels of inbreeding vary across taxa, populations, and environments, but are usually significant enough to affect both individual and population performance. In addition, data from bird and mammalian populations suggest that inbreeding depression often significantly affects birth weight, survival, reproduction, resistance to disease, predation, and environmental stress. A study conducted by Khan *et al*. [38] indicated that impaired health, fertility, and productivity of livestock species are generally considered negative effects of inbreeding. The decisions regarding mating can correct high inbreeding on farms, at least in the short-term, but long-term control of inbreeding requires consideration of the relationships between young males entering the flock [45]. The results of this study indicate that the effect of inbreeding in Lakor goats is of economic importance and is generally accepted; however, studies to document these effects are very limited. A negative effect of inbreeding depression on the loss of genetic integrity may also occur due to no selection activity [38]. Relationships among selected parents can be reduced substantially, but the corresponding reduction in genetic integrity may be large [46-48].

The level of inbreeding is an important genetic property of any population and should be determined as it can influence breeding decisions and the design of livestock improvement programs. The inbreeding process increases homozygosity for whatever genes are present, including the less desirable ones, and has been associated with a decline in livestock performance [49]. Inbreeding processes can increase homozygosity, redistribution of genetic variances, higher incidence of lethal genes, and a reduction in the performance or inbred lines. This research contributes to providing genetic information to design regulations to reduced high inbreeding depression in Lakor goats. Inbreeding depression can be minimized as a result of changes in breeding practices such as high selection intensity, use of artificial insemination, and more accurate genetic evaluation.

Crnokrak and Roff [50] reported that higher inbreeding depression occurs in the wild compared with captivity. Clearly, before reaching any general conclusion, more estimates of inbreeding depression in the wild are needed, in particular, comparisons of the same species in captivity and in the wild. The amount of inbreeding depression measured often varies according to life-history stage, traits measured, experimental habitat, environmental conditions, or year of study [44]. Increased competition, disease, or harsher field conditions can all magnify inbreeding depression [51,52]. This could put small populations exposed to both inbreeding and heightened stress at particular risk [53]. Dahlgaard and Loeschcke [54] reported that inbreeding depression is not universally higher in the wild or in more stressed populations, suggesting that we should avoid generalizations. Further data are needed to quantify how environmental and genetic factors interact to affect inbreeding depression. The results of this study indicate that inbreeding depression does occur frequently in nature, can be severe enough to affect the viability of small and isolated populations, and might often affect population dynamics.

Some studies have succeeded in searching for general patterns in the manner, in which inbreeding depression varies among taxa, environment, and population with contrasting demographics and genetic histories [50,55]. Although it is often asserted that inbreeding depression is greater under stress or field conditions, this pattern is neither universal nor theoretically resolved. It also would be of interest to know how variations in the degree of inbreeding among individuals within a population affect the expression of inbreeding depression and subsequent population dynamics. We need to learn more about how genetics and metapopulation dynamics interact, if we are to understand just when and how inbreeding contributes to the “extinction vortex” of fragmented populations. Moradi-Shaharbabak *et al*. [56] reported that in the birth records of 6598 Raeini Cashmere goats; birth weight was reduced by 6.1 g for every 1% rise in inbreeding of the inbred kids. Inbreeding in ruminants has been studied extensively and it has been concluded that inbreeding is detrimental to performance [39-41]. It was demonstrated that selection could not overcome the negative effects of inbreeding when both were practiced simultaneously, due mainly to low reproductive rates in the inbred lines [38]. The continuous rise in the level of inbreeding over the years, however, portends that future matings should be planned to avoid matings of close relatives. An increase in the number of breeding males and their frequent replacement would help reduce the level of inbreeding. In a study conducted by Khan *et al*. [38] regarding the effect of inbreeding on growth and reproduction traits of Beetal goats, pedigree records revealed that out of 4597 animals, 1966 (42.7%) were inbred, half of which had an inbreeding rate greater than 6.25%. The average inbreeding coefficient was 3.7%. There were 41 animals with inbreeding of more than 25%. About 9.1% of the animals were the result of matings, in which mates had full-sibling or stronger relationships. It is a fact that the Lakor goat population is a closed and isolated population, in which the entry of goats from outside the island does not occur. This causes a decrease in genetic diversity in the population because of inbreeding depression, death or the release of qualified Lakor goat individuals from populations that do not produce offspring due to the buying and selling process. As a result, farmers do not know the genetic information of each individual animal. However, it is still possible that there are a few individual animals in the population that have good genetic potential.

## Conclusion

This research revealed that the Lakor goat may be divided into a single clade or monophyletic group based on COI gene sequences. We found four nucleotides that can be used as genetic markers among individual animals within the Lakor goat population, *C. hircus*, and other goats based on GenBank data. The Lakor goat population has a high level of inbreeding depression as a result of geographical isolation and not allowing the introduction of males from other breeds. This has supported the formation of a monophyletic group with different genetic characteristics. Phylogenetic signals indicate that *C. aegagrus* (bezoar) is the ancestor of the native goats in Indonesia, including the Lakor goats.

## Authors’ Contributions

MR and SDV conceived the idea. RMK and SDV wrote the first draft of the manuscript. MR, RMK, SDV, and II were involved in the design and carried out the experimental work. RMK, SDV, and PK were involved in the analysis. All authors read and approved the final manuscript.
